# Removal of Upper Maxillary Bone for Myeloid Disease. Palliative Operation Two Years before

**Published:** 1866-08

**Authors:** J. Mason Warren


### REMOVAL OF UPPER MAXILLARY BONE FOR MYELOID DISEASE. PALLIATIVE OPERATION TWO YEARS BEFORE.

[Read before the Boston Society for Medical Improvement, and communicated for the Boston Medical and Surgical Journal.]

BY J. MASON WARREN, M. D.

W. A. B., a young man, 19 years of age, entered the Hospital April 7th, 1864. He was formerly a soldier. One year and a half before, a small swelling was noticed upon the outside of the gum of the upper jaw, near the first molar tooth, following an attack of typhoid fever and a severe cold in his head and face from exposure after a too early discharge from the hospital. Thinking a carious tooth in that vicinity might be the cause of the swelling, he had it removed; but the appearance of the tumor was unchanged. Twice an incision was made in it, giving exit to a quantity of blood each time. Nine months or a year after, it began to increase in size more rapidly. Upon admission to the hospital the tumor measured one and a half inches in diameter; was ovoid, hard, not tender upon pressure, painful at times, pain "streaming up" the side of the face, and at such times the eye was frequently bloodshot.

*Operation*—April 9th, the patient was etherized; the lip was drawn up, exposing the tumor. An incision was then made in the protruding wall of the tumor. Through this opening the finger was passed into a eavity containing a pulpy substance, which partly filled the antrum, and which was scooped out by the fingers. There was free hemorrhage from the opening into which a piece of sponge was inserted.

A fragment of the tumor was examined by Dr. Ellis under the microscope, and found to contain the many uncleated plates of myeloid growth, and distinct isolated nucleated cells, such as are usually found in the same connection.

Sixteen days after the operation, the patient was discharged, relieved.

On Wednesday, Nov. 29th, 1865, he returned again to the hospital for the removal of the upper maxillary, the disease having recurred and made considerable progress. Since the previous operation he had served as a soldier in the volunteer army. The right side of the cheek was now occupied by a hard tumor, projecting the whole anterior wall of the antrum, aud slightly impinging on the malar bone. The aperture mentioned in the previous account of the case was filled with a dark-colored, fungoid-looking mass, about the size of a chestnut. A consultation decided that the whole bone should be removed at once, as promising the only certain means of cure. The patient consenting, it was done in the following manner, which I shall describe in detail, as being the method which I have ordinarily persued for the removal of the upper maxillary bone, and which leaves as little deformity as any of the methods proposed, where the disease to be removed is extensive. The patient being etherized sufficiently to carry him through the preliminary incisions, a sharp-pointed bistoury was plunged through the skin just above the zygomatic process of the malar bone. A curved incision was then made through the skin and muscles to the angle of the mouth. A bit of sponge had been previously stuffed into that cheek to prevent the blood from flowing into the fauces. When the skin of the face is more flaccid, as in old persons, this incision may be commenced lower down, thereby dividing fewer filiaments of the facial nerve, leaving less paralysis. A too limited incision, however, embarrasses the section of the bones. The flap was now dissected rapidly up, the right ala of the nose cut away, and the contents of the socket dissected partially from the floor of the orbit. The bones now being well cleared, the vessels in the flaps were tied, the blood cleared away from the wound and a fresh application of ether made. With a small handsaw a groove was made in the ascending process of the malar bone and through the zygomatic process, and the section completed by the cutting forceps, the former incision extending into the sphenomaxillary fissure. The nasal process of the superior maxillary bone was now cut through in the direction of the same fissure. The mouth being held wide open, a vertical incision was made, with a strong sharp-pointed knife, through the coverings of the hard palate as far back as the palate bone, and a lateral one from the termination of this behind to the root of the last molar tooth. Liston's large cutting forceps were now used to divide the bone, which they cleanly and efficiently did, the first incisor tooth having been previously removed. The whole mass was now seized with powerful hooked forceps and an attempt made to depress it, but it held fast at its junction with the pterygoid process of the sphenoid. A chisel was therefore driven in behind the bone and an attempt made to break its attachments, but without success. A blunt chisel was then inserted between the two maxillary bones, and by a prying motion the adhesion to the bone was broken off. The remaining soft attachments were divided by curved blunt scissors. The maxillary artery spouting out freely, was restrained for a moment by means of a sponge until the fauces were freed from blood and respiration established. The vessel was then easily secured. After waiting until all hemorrhage had ceased, the edges of the skin were accurately brought together by numerous satures, great care being taken to nicely adjust the lip; and one or two satures were taken in the mucous membrane.

The patient, although a powerful young man, seemed greatly depressed from the shock of the operation, and stimulants were freely administered. There was some oozing from the wound during the day. For a number of days the pulse remained feeble and the strength much depressed. At the end of two weeks the powers of life revived, and he then began to recover with great rapidity. The paralysis of the face, which is sometimes very marked, was less than usual, and with a slight effort the eyelids could be nearly closed. Sight was not impaired. The union of the wound was perfect, except at one small spot where a salivary fistula seemed to threaten.

On inspection of the diseased part, the cavity of the antrum was found filled with a soft, dark-colored, spongy mass, which, under the microscope, presented the well-marked characters of myeloid disease. The disease was entirely removed by the operation.

*Remarks.*—The incisions made in the soft parts in this case leave as little deformity as any of those suggested for the excision of this bone. Gensoul recommends incision of the upper lip, a cross cut through the cheek and a perpendicular one at the end of this, leaving three very disagreeable scars. Fergusson, a simple cut through the upper lip into the nostril, or possibly a continuation of this incision around the margin of the ala and up the side of the nose; bpt these would hardly give room in a case like the present. In regard to the bones, it has been recommended to make one cut through the maxillary process of the malar bone into the spheno-maxillary fissure, instead of dividing the zygoma and the frontal process of the malar bone. The objection is that the development of the tumor generally occupies the whole cheek and prevents, in most cases, the execution of the plan proposed. The trying point of the operation is the adhesion of the maxillary bone to the pterygoid, made more firm by inflammatory action, so that in malignant diseases, in attempting depression of the bone, the front part of the antrum is apt to break away from the posterior portion, requiring the back part to be subsequently removed with the chisel or forceps.

The little deformity left from so very great an operation is remarkable. There is a slight paralysis in the cheek, and at first a confusion in the speech and some difficulty in deglutition, which can almost completely be remedied by artificial appliances of gold, gutta percha, or hard India rubber.

With a little management, a much larger portion of the soft parts covering the palate might be saved, as they peel off easily when the bone is depressed.

This patient recovered perfectly. The salivary fistula, which it was feared would be permanent, closed after two or three free applications of caustic. The voice and deglutition were, of course, very much impaired by the great cavity left after the removal of the bone. These, however, were completely restored by the ingenious construction of a hard rubber obturator and palate, made by Dr. Rufus E. Dickson, dentist. He now speaks well; liquids no longer regurgitate through the nostrils, and on the first of March he left for his home in New York. He was previously present and examined by the members at one of the meetings of the Boston Society for Medical Improvement, and the pathological specimen exhibited at the same time.

